# PFAPA syndrome: a review on treatment and outcome

**DOI:** 10.1186/s12969-016-0101-9

**Published:** 2016-06-27

**Authors:** Federica Vanoni, Katerina Theodoropoulou, Michaël Hofer

**Affiliations:** Pediatric Department of Southern Switzerland, Ospedale Regionale di Bellinzona e Valli, Bellinzona, 6500 Switzerland; Unité Romande d’Immuno-rhumatologie Pédiatrique (URIRP), Département Femme-Mère-Enfant (DFME), CHUV, University of Lausanne, Rue du Bugnon 46, Lausanne, 1011 Switzerland; Unité Romande d’Immuno-rhumatologie Pédiatrique (URIRP), Département Médico - Chirurgical de Pédiatrie (DFME), CHUV, University of Lausanne, Rue du Bugnon 46, Lausanne, 1011 Switzerland

**Keywords:** PFAPA treatment, PFAPA outcome, Tonsillectomy, Glucocorticoids, Colchicine

## Abstract

The syndrome of periodic fever, aphthous stomatitis, pharyngitis and cervical adenitis (PFAPA syndrome) is the most common cause of periodic fever in childhood. The current pharmacological treatment includes corticosteroids, which usually are efficacious in the management of fever episodes, colchicine, for the prophylaxis of febrile episodes, and other medication for which efficacy has not been proven so far. Tonsillectomy is an option for selected patients. Usually PFAPA syndrome resolves during adolescence, but there is increasing evidence that this condition may persist into adulthood.

## Background

The syndrome of periodic fever, aphthous stomatitis, pharyngitis and cervical adenitis (PFAPA syndrome) is the most common cause of periodic fever in childhood and it was first described in1987 by Marshall et al. [1]. It is characterized by episodes of fever lasting for 3–6 days with recurrence every 3–8 weeks, associated with at least one of three main symptoms: aphthous stomatitis, cervical adenitis, and pharyngitis [2]. Disease onset is usually before the age of 5 and generally resolves by adolescence. Patients are asymptomatic between episodes and show normal growth. Proposed contributors to pathogenesis include infection, abnormal host immune responses, or a combination of both [3, 4]. PFAPA syndrome is an immune mediated disease characterised by a cytokine dysfunction [3, 5]; moreover, the strong familial clustering suggest a potential genetic origin of the syndrome [6, 7]. The presence of variants in inflammasome related genes, mostly in *NLRP3* and *MEFV*, suggest a possible role of these genes in PFAPA pathogenesis [7–9]. However, none of these variants alone seem to be relevant for the disease etiology, suggesting an oligenic or polygenic background.

Currently the diagnosis of PFAPA is based on clinical criteria [2] (Table 1), but these criteria have not been validated in a cohort of patients. Moreover, Gattorno et al. found that a significant number of patients with monogenic periodic fevers also meet the diagnostic criteria for PFAPA syndrome [10], highlighting the poor specificity of the current classification criteria. Therefore, patients should be screened clinically or genetically for other known periodic syndromes before assigning the diagnosis of PFAPA.

**Table 1:** Diagnostic criteria used for PFAPA

I. Regularly recurring fevers with an early age of onset (<5 years of age)
II. Constitutional symptoms in the absence of upper respiratory infection with at least 1 of the following clinical signs : a) aphtous stomatitis, b) cervical lymphadenitis, c) pharyngitis
III. Completely asymptomatic interval between episodes
IV. Normal growth and development

PFAPA syndrome has favourable natural history. There is no evidence that medical treatment can modify the outcome, but it can be efficacious for treating the episodes (Table 2). Inducing a rapid remission of episodes is important to improve the quality of life for patients and their families. In this paper we review the current treatment strategies for PFAPA and what is known about the outcome of this syndrome.

**Table 2:** Pharmacological treatment for PFAPA syndrome

Treatment of the episodes		
	Dose	Remarks
Prednisone	0.5–2 mg/Kg, orally the first day of fever	Possible to repeat on day 2 if fever persists
Betametasone	0.2 mg/Kg, orally the first day of fever	Possible to repeat on day 2 if fever persists
Prophylactic Treatment		
	Dose	Remarks
Colchicine	0.5–1 mg/daily, orally	Gastro-intestinal side effects
Cimetidine	20–40/mg/Kg/daily, orally	Poor efficacy
Anakinra	1 mg/Kg, sc the first and second day of fever	Cost-effectiveness

### Symptomatic treatment during flares

Non-steroidal anti-inflammatory agents and anti-pyretics have shown poor results in resolving symptoms of PFAPA syndrome. Glucocorticoids are highly effective in aborting the attacks, but there are limited data on the effectiveness of any preventive medication in PFAPA.

### Rapid resolution of flare: glucocorticoids

Orally given glucocorticoids relieve symptoms of PFAPA in a dramatic fashion [11–15].

A single dose of prednisone (1– 2 mg/kg) or betamethasone (0.1–0.2 mg/kg) given at the onset of an episode can dramatically abort fever attacks in a few hours. Aphtous stomatitis, however, can take longer to resolve [2, 16]. If one dose is not effective in inducing resolution of flare, a second dose may be given the following day.

In the largest PFAPA cohort described so far, Hofer et al. found that 147 out of 301 patients were treated with steroids. They observed a rapid resolution of fever episodes after a single dose of steroids in 93/147 patients (63 %), whereas 46 (32 %) showed a partial response and only 8 (5 %) were non-responders [16]. Data from the EUROFEVER registry confirm the widespread use of steroids for disease flare, with 81 out of 92 patients treated at the onset of attacks, and their effectiveness in 73 patients (90 % of those treated) [13]. Wurster et al. described a cohort of 60 patients in which 44 patients were treated with steroids during episodes and the treatment was effective in 37 (84 %) [17].

Tasher et al., in their uncontrolled series, described that a single low dose of prednisone (mean 0.6 mg/kg per day) was effective to rapidly resolve the fever episode within an average of 10 h in 51 out of 54 PFAPA patients [18]. The effectiveness of a single low dose of prednisone was confirmed in a preliminary study performed by Yazgan et al., that did not show any statistical significance in efficacy between a dose of 2 mg/kg/day versus a dose of 0.5 mg/kg/day respectively in 40 and 46 PFAPA febrile attacks [19].

The usefulness of steroids is limited by the fact that the interval between episodes may be shortened in 25–50 % of cases [15, 20, 21]. Moreover, the administration of corticosteroids does not prevent future fever attacks. Side effects are rare; the most commonly reported by Tasher et al. is restlessness. Nevertheless, parents of PFAPA patients are often concerned about the possibility of systemic side effects, and this fact may result in poor compliance.

Finally, the steroid response may be useful in distinguishing attacks of PFAPA from familial Mediterranean fever (FMF) or other hereditary periodic fever syndromes (HPF) [11, 12] and can be used as an additional criterion for diagnosis [16]. In fact, HPF attack, except for periodic fever associated with Mevalonate Kinase Deficiency (MKD), usually doesn’t show a dramatic response to a single dose of steroids like PFAPA.

### Reduction of flare frequency: colchicine

The precise mechanism of action of colchicine in reducing inflammation is unknown. Colchicine binds to tubulin, forming a tubulin-colchicine complex. This complex can change the structure and the function of the cytoskeleton, thereby influencing neutrophil and lymphocyte migration and adhesion [22]. The rationale for the use of colchicine as a prophylactic treatment for PFAPA is based primarily on clinical and laboratory similarities between FMF and PFAPA and the long-term experience with this drug in the treatment of FMF. For these reasons, when colchicine is effective in PFAPA patients, an alternative diagnosis of FMF has to be considered.

In a 6-month open label, randomized, controlled study, Aviel et al. showed a significant increase in intervals between attacks in 8 PFAPA patients on colchicine therapy compared with 10 patients treated only with corticosteroids. Among the 18 treated patients treated, 8 carried FMF mutations; 6/8 in the colchicine group and 2/10 in the steroid group [23].

Padeh et al. described 10 PFAPA patients previously diagnosed as FMF patients; 6 out of 10 were heterozygous for an *MEFV* gene mutation (M694V), but had clinical features consistent with PFAPA. Colchicine prescribed to these patients had only a partial effect and was discontinued [24].

In an open label study, Tasher et al. investigated the efficacy of colchicine treatment in 9 PFAPA patients with frequent episodes (≤14 days interval). Two patients out of nine were compound heterozygotes for multiple *MEFV* mutations but exhibited typical features of PFAPA. Colchicine treatment significantly increased the interval between the episodes in eight of these patients [25].

Dusser et al. performed a retrospective, multicenter study in which they reviewed 20 PFAPA patients treated with colchicine. Half of the patients were heterozygous for a pathogenic mutation in the *MEFV* gene. The authors found that nine patients had no more or suffered half as many episodes during colchicine treatment. No significant differences in demographic/clinical variables or the rate of *MEFV* mutation carriage were found between the two groups (responder and non-responder) [26].

Due to the small size of the samples, Aviel and Dusser didn’t find variables that could predict responsiveness to colchicine. Because of the good response of FMF to colchicine, PFAPA patients heterozygous for *MEFV* mutations may respond better to this drug, but differences in the response to colchicine between carriers and non-carriers of the *MEFV* mutation have not been demonstrated so far.

Colchicine is usually well tolerated. The most common adverse reactions to colchicine are gastrointestinal (approximately 10 %). This effect may be partially explained by induction or worsening of lactose intolerance by this medication [25], though multiple mechanism may be responsible.

These findings suggest that colchicine may be an effective second line treatment to prevent frequently recurrent fever episodes in PFAPA patients, in particular if prednisone is decreases the interval between episodes.

### Other medications

Cimetidine, a common H2 antagonist, has immune-modulating properties, inhibiting chemotaxis and T-cell activation. Cimetidine was suggested as an effective prophylactic treatment for PFAPA in 1992 by Feder [27]. Thomas et al. reported a 43 % efficacy of cimetidine in a group of 28 patients according to data based on telephone recall [2]. Wurster et al. found that in their cohort, cimetidine was effective as symptomatic therapy in 6 out of 25 (26 %) patients while in the remaining patients the treatment was ineffective [17]. None of the 92 PFAPA patients from the EUROFEVER registry, and none of the 42 patients from the Norwegian cohort, were treated with cimetidine, underlining the fact that in recent years this treatment is less prescribed [13, 21]. Moreover, there are no randomized controlled trials supporting benefits of cimetidine to date.

IL-1 plays a central role in PFAPA pathogenesis, as demonstrated by Stojanov et al. [3]. In a small cohort of 5 children with PFAPA syndrome a single dose of anakinra, on the second day of fever, dramatically improved both clinical picture and laboratory parameters [5]. Cantarini et al. described a case of a 27-year-old man resistant to conventional therapy (corticosteroids, colchicine, and tonsillectomy) who was treated with subcutaneous injection of anakinra, with a complete resolution of fever attacks [28]. Despite these interesting reports, the use of IL-1 blockers for PFAPA treatment is restricted to selected cases due to the lack of both randomized trials as well as coverage by health care plans.

Vitamin D has recently gained attention as a possible regulator of inflammation due to the finding that low Vitamin D levels are associated with some inflammatory disorders [29]. In recent years two studies investigated a possible role of vitamin D in PFAPA syndrome. Mahamid et al. found a significant correlation between PFAPA and vitamin D deficiency, demonstrating a significant difference in vitamin D levels between 22 PFAPA patients and 20 control subjects [30]. Stagi et al. confirmed this finding and demonstrated a significant reduction in the number of febrile episodes and a shortening of mean duration of episodes in patients after vitamin D supplementation (400 IU 25-hydroxyvitamin D daily during wintertime) [31]. However, on the basis of these data it is not possible to conclude that vitamin D is effective in treating or preventing PFAPA syndrome, and prospective studies on large cohorts of patients and randomized clinical trials are needed.

### Adenotonsillectomy

The role of tonsillectomy in PFAPA syndrome remains controversial. In 1989 an initial study reported efficacy of tonsillectomy in 4 PFAPA patients [32]. Afterwards, a large variability in success rate was reported in other studies. In a randomized controlled trial, Renko et al. compared the effectiveness of tonsillectomy versus no intervention in 26 patients diagnosed with PFAPA (14 underwent tonsillectomy and 12 were observed without surgery) [33]. PFAPA syndrome resolved immediately in all 14 patients randomized to tonsillectomy; in contrast, the syndrome resolved spontaneously within 6 months in 6/12 patients who did not receive tonsillectomy. However, in this cohort, 50 % of the tonsillectomy group and 77 % of the control group had recurrent fever as the only cardinal symptom and thereby did not actually fulfil diagnostic criteria for PFAPA.

Garavello et al. performed a prospective randomized controlled trial with 39 PFAPA patients; 19 underwent adenotonsillectomy and 20 were treated with medical therapy [34]. Six months after surgery, resolution of episodes was observed in 12/19 cases (63 %) and at 18 months follow up they observed complete resolution of episodes in this group within 1 year. In contrast to the findings of Renko et al., only 1 patient in the control group showed spontaneous resolution.

A prospective study by Licameli et al. evaluated the long-term efficacy of adeno-tonsillectomy in 102 patients with PFAPA followed for longer than 6 months after surgery (mean 43 months): Ninety-nine experienced complete resolution immediately aſter surgery and 1 after 6 months. Of the 2 remaining patients, 1 continued having fever episodes, while 1 was further investigated and subsequently diagnosed with MKD [35].

Although other reports exist, a recent Cochrane review [36] points out that only two randomised controlled trials, conducted on small cohorts of patients, demonstrate the effectiveness of the tonsillectomy in treating children with PFAPA. Moreover, these studies show some differences in outcome after surgery, probably due to the heterogeneity of the study population, the choice of different diagnostic criteria, the different type of intervention (i.e., tonsillectomy versus adenotonsillectomy), and the different follow up schedule aſter surgery. It is not proven that to combining adenoidectomy with tonsillectomy can improve the outcome, compared to tonsillectomy alone.

Considering the favourable evolution of PFAPA and the possible post-surgical complications, adenotonsillectomy should be proposed to selected patients, for example when the interval between attacks is very short in which corticosteroid treatment is not appropriate.

### Outcome

PFAPA syndrome is considered a self-limited disease that generally resolves spontaneously before adolescence [16, 17]. Patients’ growth and development are normal and no long-term consequences have been described [2].

Wurster et al. followed a cohort of 59 patients for a period ranging between 12 and 21 years. Fifty patients experienced spontaneous symptom resolution without relapse; only 9 maintained typical cardinal PFAPA symptoms, though fever was less frequent [17]. They also observed a significantly higher frequency of family history of periodic fever in the patients with persistent symptoms in adulthood.

In a Norwegian cohort, thirty-seven children were followed until resolution with a median follow up of 18.7 months (range 7.2– 75.7). The median age at the time of resolution was 52.1 months. Interestingly, eight children experienced a relapse after a febrile attack-free period of more than 6 months. The median duration of the attack-free periods leading up to the relapse was 20 months [21].

Other studies, with a shorter follow-up, reported a spontaneous resolution in only 20–32 % of the patients [12, 24, 25].

Berkun et al. described a cohort of 124 PFAPA patients, of which 65 showed a single *MEFV* mutation. PFAPA attacks in *MEFV* carriers were shorter, compared with patients without mutations, and the frequency of their attacks and oral aphthae were also lower. This may suggest that mutations in causative genes of other monogenic periodic fevers may modify the disease course [37].

As shown in different studies, PFAPA may have its onset in adulthood as well [11, 16, 38]. To date, no long-term outcome data are available for adult patients diagnosed with PFAPA syndrome, so it is not known whether adults with PFAPA syndrome may spontaneously undergo clinical remission. Based on a review of the recent literature, tonsillectomy does not seem to be a valid option in these patients [39]. There are reports describing adult PFAPA patients with a history of tonsillectomy during childhood due to recurrent febrile tonsillo-pharyngitis, with subsequent disease free period of several years. These findings may suggest that tonsillectomy is efficacious in inducing a temporary remission but that the effect may be transient [40].

## Conclusions

PFAPA syndrome is a relatively common condition in childhood, but it can also persist into adulthood. The disease usually has a benign and self-limiting course. Treatment with single doses of glucocorticoids is effective in controlling the fever episodes. Colchicine may be an interesting option for patients with frequent episodes. Tonsillectomy should be reserved for selected patients refractory to medical treatment and children with long-lasting disease affecting the quality of life.

## Abbreviations

FMF, familial mediterranean fever; HFP, hereditary periodic fever; MKD, mevalonate kinase deficiency; PFAPA, periodic fever, aphthous stomatitis, cervical adenitis
